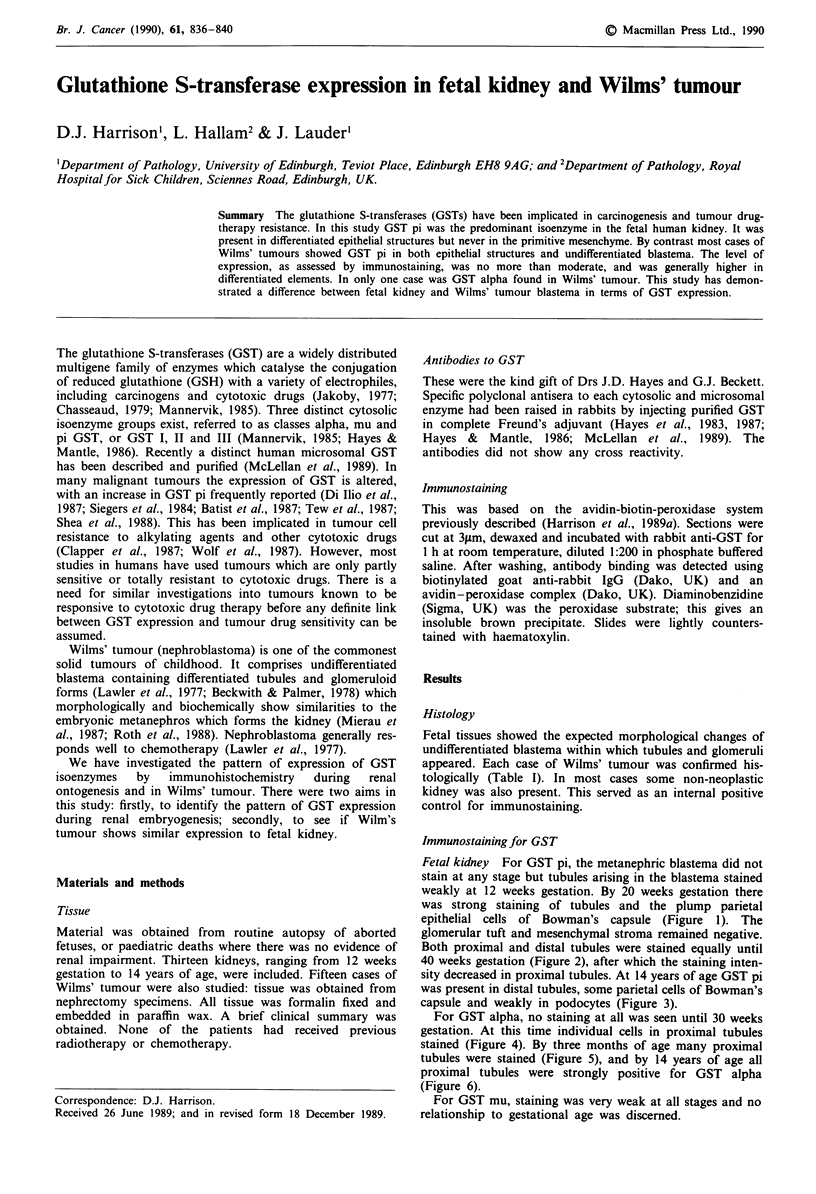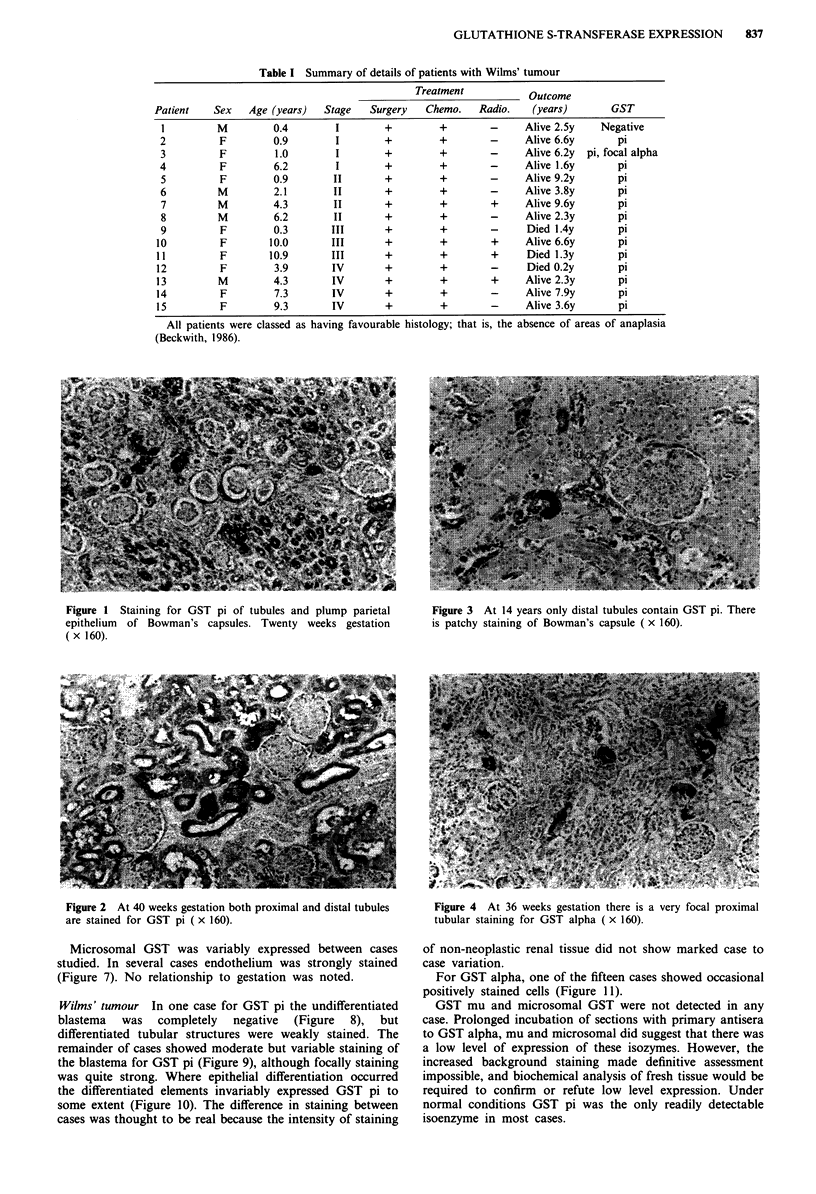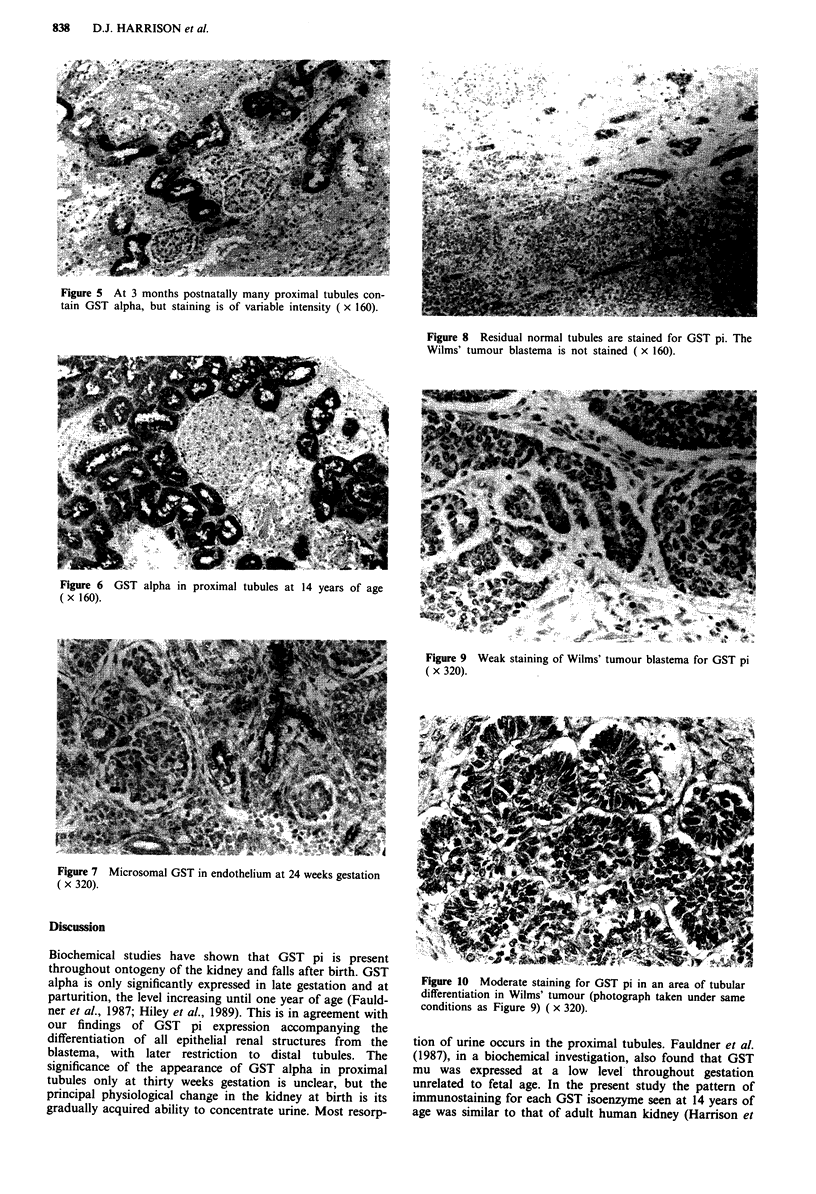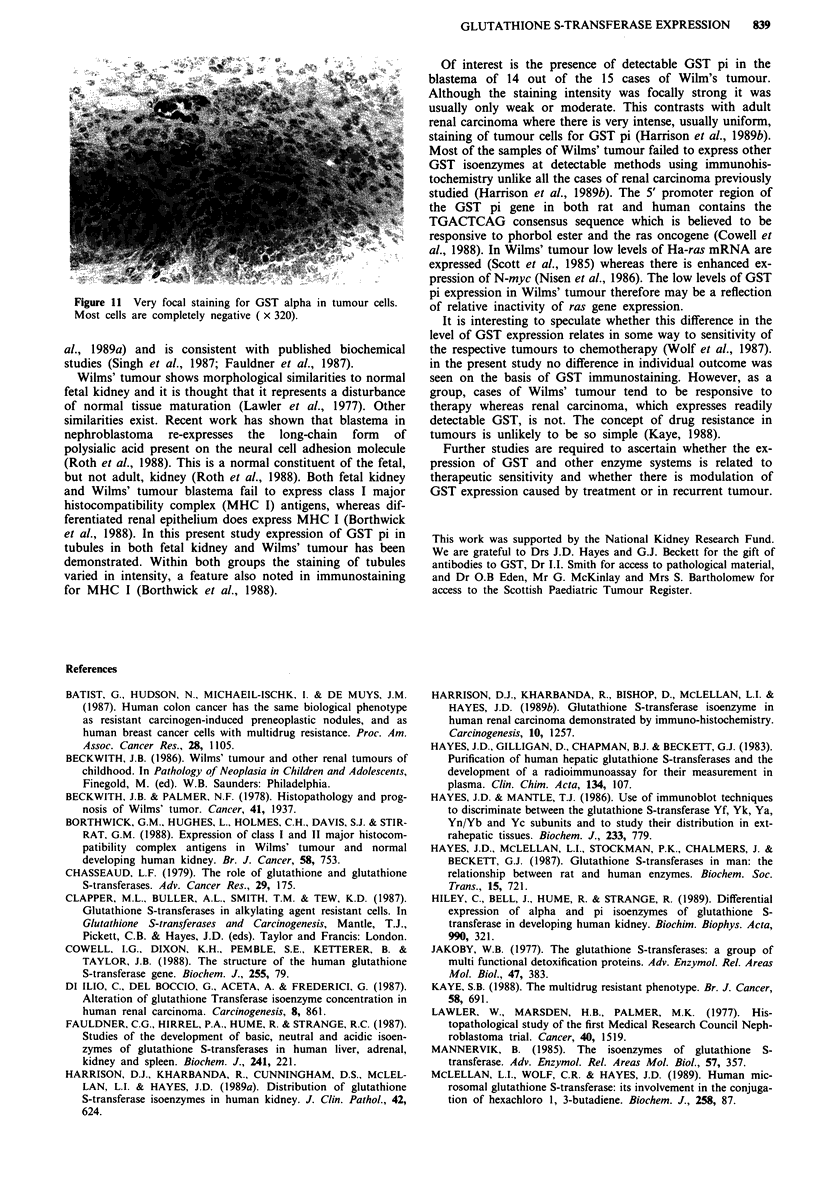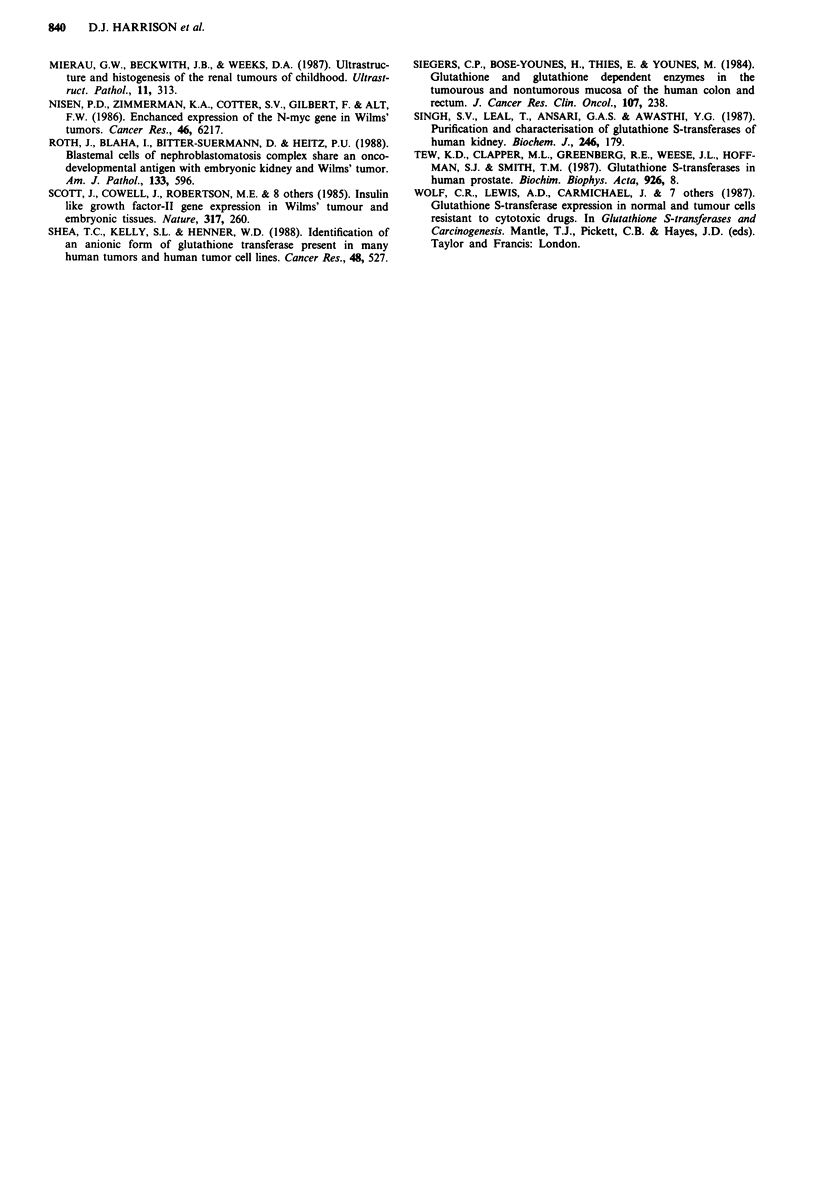# Glutathione S-transferase expression in fetal kidney and Wilms' tumour.

**DOI:** 10.1038/bjc.1990.187

**Published:** 1990-06

**Authors:** D. J. Harrison, L. Hallam, J. Lauder

**Affiliations:** Department of Pathology, University of Edinburgh, UK.

## Abstract

**Images:**


Br. J. Cancer (1990), 61, 836-840

© Macmillan Press Ltd., 1990

### Glutathione S-transferase expression in fetal kidney and Wilms' tumour

D.J. Harrison[1], L. Hallam[2] & J. Lauder[1]

[1]Department of Pathology, University of Edinburgh, Teviot Place, Edinburgh EH8 9AG; and [2]Department of Pathology, Royal Hospital for Sick Children, Sciennes Road, Edinburgh, UK.

**Summary** The glutathione S-transferases (GSTs) have been implicated in carcinogenesis and tumour drug-therapy resistance. In this study GST pi was the predominant isoenzyme in the fetal human kidney. It was present in differentiated epithelial structures but never in the primitive mesenchyme. By contrast most cases of Wilms' tumours showed GST pi in both epithelial structures and undifferentiated blastema. The level of expression, as assessed by immunostaining, was no more than moderate, and was generally higher in differentiated elements. In only one case was GST alpha found in Wilms' tumour. This study has demonstrated a difference between fetal kidney and Wilms' tumour blastema in terms of GST expression.

The glutathione S-transferases (GST) are a widely distributed multigene family of enzymes which catalyse the conjugation of reduced glutathione (GSH) with a variety of electrophiles, including carcinogens and cytotoxic drugs (Jakoby, 1977; Chasseaud, 1979; Mannervik, 1985). Three distinct cytosolic isoenzyme groups exist, referred to as classes alpha, mu and pi GST, or GST I, II and III (Mannervik, 1985; Hayes & Mantle, 1986). Recently a distinct human microsomal GST has been described and purified (McLellan *et al.*, 1989). In many malignant tumours the expression of GST is altered, with an increase in GST pi frequently reported (Di Ilio *et al.*, 1987; Siegers *et al.*, 1984; Batist *et al.*, 1987; Tew *et al.*, 1987; Shea *et al.*, 1988). This has been implicated in tumour cell resistance to alkylating agents and other cytotoxic drugs (Clapper *et al.*, 1987; Wolf *et al.*, 1987). However, most studies in humans have used tumours which are only partly sensitive or totally resistant to cytotoxic drugs. There is a need for similar investigations into tumours known to be responsive to cytotoxic drug therapy before any definite link between GST expression and tumour drug sensitivity can be assumed.

Wilms' tumour (nephroblastoma) is one of the commonest solid tumours of childhood. It comprises undifferentiated blastema containing differentiated tubules and glomeruloid forms (Lawler *et al.*, 1977; Beckwith & Palmer, 1978) which morphologically and biochemically show similarities to the embryonic metanephros which forms the kidney (Mierau *et al.*, 1987; Roth *et al.*, 1988). Nephroblastoma generally responds well to chemotherapy (Lawler *et al.*, 1977).

We have investigated the pattern of expression of GST isoenzymes by immunohistochemistry during renal ontogenesis and in Wilms' tumour. There were two aims in this study: firstly, to identify the pattern of GST expression during renal embryogenesis; secondly, to see if Wilm's tumour shows similar expression to fetal kidney.

### Materials and methods

### *Tissue*

Material was obtained from routine autopsy of aborted fetuses, or paediatric deaths where there was no evidence of renal impairment. Thirteen kidneys, ranging from 12 weeks gestation to 14 years of age, were included. Fifteen cases of Wilms' tumour were also studied: tissue was obtained from nephrectomy specimens. All tissue was formalin fixed and embedded in paraffin wax. A brief clinical summary was obtained. None of the patients had received previous radiotherapy or chemotherapy.

Correspondence: D.J. Harrison.

Received 26 June 1989; and in revised form 18 December 1989.

### *Antibodies to GST*

These were the kind gift of Drs J.D. Hayes and G.J. Beckett. Specific polyclonal antisera to each cytosolic and microsomal enzyme had been raised in rabbits by injecting purified GST in complete Freund's adjuvant (Hayes *et al.*, 1983, 1987; Hayes & Mantle, 1986; McLellan *et al.*, 1989). The antibodies did not show any cross reactivity.

### *Immunostaining*

This was based on the avidin-biotin-peroxidase system previously described (Harrison *et al.*, 1989*a*). Sections were cut at 3μm, dewaxed and incubated with rabbit anti-GST for 1 h at room temperature, diluted 1:200 in phosphate buffered saline. After washing, antibody binding was detected using biotinylated goat anti-rabbit IgG (Dako, UK) and an avidin-peroxidase complex (Dako, UK). Diaminobenzidine (Sigma, UK) was the peroxidase substrate; this gives an insoluble brown precipitate. Slides were lightly counterstained with haematoxylin.

### Results

### *Histology*

Fetal tissues showed the expected morphological changes of undifferentiated blastema within which tubules and glomeruli appeared. Each case of Wilms' tumour was confirmed histologically (Table I). In most cases some non-neoplastic kidney was also present. This served as an internal positive control for immunostaining.

### *Immunostaining for GST*

*Fetal kidney* For GST pi, the metanephric blastema did not stain at any stage but tubules arising in the blastema stained weakly at 12 weeks gestation. By 20 weeks gestation there was strong staining of tubules and the plump parietal epithelial cells of Bowman's capsule (Figure 1). The glomerular tuft and mesenchymal stroma remained negative. Both proximal and distal tubules were stained equally until 40 weeks gestation (Figure 2), after which the staining intensity decreased in proximal tubules. At 14 years of age GST pi was present in distal tubules, some parietal cells of Bowman's capsule and weakly in podocytes (Figure 3).

For GST alpha, no staining at all was seen until 30 weeks gestation. At this time individual cells in proximal tubules stained (Figure 4). By three months of age many proximal tubules were stained (Figure 5), and by 14 years of age all proximal tubules were strongly positive for GST alpha (Figure 6).

For GST mu, staining was very weak at all stages and no relationship to gestational age was discerned.

**Table I** Summary of details of patients with Wilms' tumour

| *Patient* | *Sex* | *Age (years)* | *Stage* | *Treatment* | | | *Outcome (years)* | *GST* |
|---|---|---|---|---|---|---|---|---|
| | | | | *Surgery* | *Chemo.* | *Radio.* | | |
| 1 | M | 0.4 | I | + | + | – | Alive 2.5y | Negative |
| 2 | F | 0.9 | I | + | + | – | Alive 6.6y | pi |
| 3 | F | 1.0 | I | + | + | – | Alive 6.2y | pi, focal alpha |
| 4 | F | 6.2 | I | + | + | – | Alive 1.6y | pi |
| 5 | F | 0.9 | II | + | + | – | Alive 9.2y | pi |
| 6 | M | 2.1 | II | + | + | – | Alive 3.8y | pi |
| 7 | M | 4.3 | II | + | + | + | Alive 9.6y | pi |
| 8 | M | 6.2 | II | + | + | – | Alive 2.3y | pi |
| 9 | F | 0.3 | III | + | + | – | Died 1.4y | pi |
| 10 | F | 10.0 | III | + | + | + | Alive 6.6y | pi |
| 11 | F | 10.9 | III | + | + | + | Died 1.3y | pi |
| 12 | F | 3.9 | IV | + | + | – | Died 0.2y | pi |
| 13 | M | 4.3 | IV | + | + | + | Alive 2.3y | pi |
| 14 | F | 7.3 | IV | + | + | – | Alive 7.9y | pi |
| 15 | F | 9.3 | IV | + | + | – | Alive 3.6y | pi |

All patients were classed as having favourable histology; that is, the absence of areas of anaplasia (Beckwith, 1986).

**Figure 1** Staining for GST pi of tubules and plump parietal epithelium of Bowman's capsules. Twenty weeks gestation ( × 160).

**Figure 3** At 14 years only distal tubules contain GST pi. There is patchy staining of Bowman's capsule ( × 160).

**Figure 2** At 40 weeks gestation both proximal and distal tubules are stained for GST pi ( × 160).

**Figure 4** At 36 weeks gestation there is a very focal proximal tubular staining for GST alpha ( × 160).

Microsomal GST was variably expressed between cases studied. In several cases endothelium was strongly stained (Figure 7). No relationship to gestation was noted.

*Wilms' tumour* In one case for GST pi the undifferentiated blastema was completely negative (Figure 8), but differentiated tubular structures were weakly stained. The remainder of cases showed moderate but variable staining of the blastema for GST pi (Figure 9), although focally staining was quite strong. Where epithelial differentiation occurred the differentiated elements invariably expressed GST pi to some extent (Figure 10). The difference in staining between cases was thought to be real because the intensity of staining of non-neoplastic renal tissue did not show marked case to case variation.

For GST alpha, one of the fifteen cases showed occasional positively stained cells (Figure 11).

GST mu and microsomal GST were not detected in any case. Prolonged incubation of sections with primary antisera to GST alpha, mu and microsomal did suggest that there was a low level of expression of these isozymes. However, the increased background staining made definitive assessment impossible, and biochemical analysis of fresh tissue would be required to confirm or refute low level expression. Under normal conditions GST pi was the only readily detectable isoenzyme in most cases.

**Figure 5** At 3 months postnatally many proximal tubules contain GST alpha, but staining is of variable intensity ( × 160).

**Figure 6** GST alpha in proximal tubules at 14 years of age ( × 160).

**Figure 7** Microsomal GST in endothelium at 24 weeks gestation ( × 320).

### Discussion

Biochemical studies have shown that GST pi is present throughout ontogeny of the kidney and falls after birth. GST alpha is only significantly expressed in late gestation and at parturition, the level increasing until one year of age (Fauldner *et al.*, 1987; Hiley *et al.*, 1989). This is in agreement with our findings of GST pi expression accompanying the differentiation of all epithelial renal structures from the blastema, with later restriction to distal tubules. The significance of the appearance of GST alpha in proximal tubules only at thirty weeks gestation is unclear, but the principal physiological change in the kidney at birth is its gradually acquired ability to concentrate urine. Most resorp-

**Figure 8** Residual normal tubules are stained for GST pi. The Wilms' tumour blastema is not stained ( × 160).

**Figure 9** Weak staining of Wilms' tumour blastema for GST pi ( × 320).

**Figure 10** Moderate staining for GST pi in an area of tubular differentiation in Wilms' tumour (photograph taken under same conditions as Figure 9) ( × 320).

tion of urine occurs in the proximal tubules. Fauldner *et al.* (1987), in a biochemical investigation, also found that GST mu was expressed at a low level throughout gestation unrelated to fetal age. In the present study the pattern of immunostaining for each GST isoenzyme seen at 14 years of age was similar to that of adult human kidney (Harrison *et*

**Figure 11** Very focal staining for GST alpha in tumour cells. Most cells are completely negative ( × 320).

*al.*, 1989*a*) and is consistent with published biochemical studies (Singh *et al.*, 1987; Fauldner *et al.*, 1987).

Wilms' tumour shows morphological similarities to normal fetal kidney and it is thought that it represents a disturbance of normal tissue maturation (Lawler *et al.*, 1977). Other similarities exist. Recent work has shown that blastema in nephroblastoma re-expresses the long-chain form of polysialic acid present on the neural cell adhesion molecule (Roth *et al.*, 1988). This is a normal constituent of the fetal, but not adult, kidney (Roth *et al.*, 1988). Both fetal kidney and Wilms' tumour blastema fail to express class I major histocompatibility complex (MHC I) antigens, whereas differentiated renal epithelium does express MHC I (Borthwick *et al.*, 1988). In this present study expression of GST pi in tubules in both fetal kidney and Wilms' tumour has been demonstrated. Within both groups the staining of tubules varied in intensity, a feature also noted in immunostaining for MHC I (Borthwick *et al.*, 1988).

Of interest is the presence of detectable GST pi in the blastema of 14 out of the 15 cases of Wilm's tumour. Although the staining intensity was focally strong it was usually only weak or moderate. This contrasts with adult renal carcinoma where there is very intense, usually uniform, staining of tumour cells for GST pi (Harrison *et al.*, 1989*b*). Most of the samples of Wilms' tumour failed to express other GST isoenzymes at detectable methods using immunohistochemistry unlike all the cases of renal carcinoma previously studied (Harrison *et al.*, 1989*b*). The 5′ promoter region of the GST pi gene in both rat and human contains the TGACTCAG consensus sequence which is believed to be responsive to phorbol ester and the ras oncogene (Cowell *et al.*, 1988). In Wilms' tumour low levels of Ha-*ras* mRNA are expressed (Scott *et al.*, 1985) whereas there is enhanced expression of N-*myc* (Nisen *et al.*, 1986). The low levels of GST pi expression in Wilms' tumour therefore may be a reflection of relative inactivity of *ras* gene expression.

It is interesting to speculate whether this difference in the level of GST expression relates in some way to sensitivity of the respective tumours to chemotherapy (Wolf *et al.*, 1987). in the present study no difference in individual outcome was seen on the basis of GST immunostaining. However, as a group, cases of Wilms' tumour tend to be responsive to therapy whereas renal carcinoma, which expresses readily detectable GST, is not. The concept of drug resistance in tumours is unlikely to be so simple (Kaye, 1988).

Further studies are required to ascertain whether the expression of GST and other enzyme systems is related to therapeutic sensitivity and whether there is modulation of GST expression caused by treatment or in recurrent tumour.

This work was supported by the National Kidney Research Fund. We are grateful to Drs J.D. Hayes and G.J. Beckett for the gift of antibodies to GST, Dr I.I. Smith for access to pathological material, and Dr O.B Eden, Mr G. McKinlay and Mrs S. Bartholomew for access to the Scottish Paediatric Tumour Register.

### References

BATIST, G., HUDSON, N., MICHAEIL-ISCHK, I. & DE MUYS, J.M. (1987). Human colon cancer has the same biological phenotype as resistant carcinogen-induced preneoplastic nodules, and as human breast cancer cells with multidrug resistance. *Proc. Am. Assoc. Cancer Res.*, **28**, 1105.

BECKWITH, J.B. (1986). Wilms' tumour and other renal tumours of childhood. In *Pathology of Neoplasia in Children and Adolescents*, Finegold, M. (ed). W.B. Saunders: Philadelphia.

BECKWITH, J.B. & PALMER, N.F. (1978). Histopathology and prognosis of Wilms' tumor. *Cancer*, **41**, 1937.

BORTHWICK, G.M., HUGHES, L., HOLMES, C.H., DAVIS, S.J. & STIRRAT, G.M. (1988). Expression of class I and II major histocompatibility complex antigens in Wilms' tumour and normal developing human kidney. *Br. J. Cancer*, **58**, 753.

CHASSEAUD, L.F. (1979). The role of glutathione and glutathione S-transferases. *Adv. Cancer Res.*, **29**, 175.

CLAPPER, M.L., BULLER, A.L., SMITH, T.M. & TEW, K.D. (1987). Glutathione S-transferases in alkylating agent resistant cells. In *Glutathione S-transferases and Carcinogenesis*, Mantle, T.J., Pickett, C.B. & Hayes, J.D. (eds). Taylor and Francis: London.

COWELL, I.G., DIXON, K.H., PEMBLE, S.E., KETTERER, B. & TAYLOR, J.B. (1988). The structure of the human glutathione S-transferase gene. *Biochem. J.*, **255**, 79.

DI ILIO, C., DEL BOCCIO, G., ACETA, A. & FREDERICI, G. (1987). Alteration of glutathione Transferase isoenzyme concentration in human renal carcinoma. *Carcinogenesis*, **8**, 861.

FAULDNER, C.G., HIRREL, P.A., HUME, R. & STRANGE, R.C. (1987). Studies of the development of basic, neutral and acidic isoenzymes of glutathione S-transferases in human liver, adrenal, kidney and spleen. *Biochem. J.*, **241**, 221.

HARRISON, D.J., KHARBANDA, R., CUNNINGHAM, D.S., MCLELLAN, L.I. & HAYES, J.D. (1989*a*). Distribution of glutathione S-transferase isoenzymes in human kidney. *J. Clin. Pathol.*, **42**, 624.

HARRISON, D.J., KHARBANDA, R., BISHOP, D., MCLELLAN, L.I. & HAYES, J.D. (1989*b*). Glutathione S-transferase isoenzyme in human renal carcinoma demonstrated by immuno-histochemistry. *Carcinogenesis*, **10**, 1257.

HAYES, J.D., GILLIGAN, D., CHAPMAN, B.J. & BECKETT, G.J. (1983). Purification of human hepatic glutathione S-transferases and the development of a radioimmunoassay for their measurement in plasma. *Clin. Chim. Acta*, **134**, 107.

HAYES, J.D. & MANTLE, T.J. (1986). Use of immunoblot techniques to discriminate between the glutathione S-transferase Yf, Yk, Ya, Yn/Yb and Yc subunits and to study their distribution in extrahepatic tissues. *Biochem. J.*, **233**, 779.

HAYES, J.D., MCLELLAN, L.I., STOCKMAN, P.K., CHALMERS, J. & BECKETT, G.J. (1987). Glutathione S-transferases in man: the relationship between rat and human enzymes. *Biochem. Soc. Trans.*, **15**, 721.

HILEY, C., BELL, J., HUME, R. & STRANGE, R. (1989). Differential expression of alpha and pi isoenzymes of glutathione S-transferase in developing human kidney. *Biochim. Biophys. Acta*, **990**, 321.

JAKOBY, W.B. (1977). The glutathione S-transferases: a group of multi functional detoxification proteins. *Adv. Enzymol. Rel. Areas Mol. Biol.*, **47**, 383.

KAYE, S.B. (1988). The multidrug resistant phenotype. *Br. J. Cancer*, **58**, 691.

LAWLER, W., MARSDEN, H.B., PALMER, M.K. (1977). Histopathological study of the first Medical Research Council Nephroblastoma trial. *Cancer*, **40**, 1519.

MANNERVIK, B. (1985). The isoenzymes of glutathione S-transferase. *Adv. Enzymol. Rel. Areas Mol. Biol.*, **57**, 357.

MCLELLAN, L.I., WOLF, C.R. & HAYES, J.D. (1989). Human microsomal glutathione S-transferase: its involvement in the conjugation of hexachloro 1, 3-butadiene. *Biochem. J.*, **258**, 87.

MIERAU, G.W., BECKWITH, J.B., & WEEKS, D.A. (1987). Ultrastructure and histogenesis of the renal tumours of childhood. *Ultrastruct. Pathol.*, **11**, 313.

NISEN, P.D., ZIMMERMAN, K.A., COTTER, S.V., GILBERT, F. & ALT, F.W. (1986). Enchanced expression of the N-myc gene in Wilms' tumors. *Cancer Res.*, **46**, 6217.

ROTH, J., BLAHA, I., BITTER-SUERMANN, D. & HEITZ, P.U. (1988). Blastemal cells of nephroblastomatosis complex share an onco-developmental antigen with embryonic kidney and Wilms' tumor. *Am. J. Pathol.*, **133**, 596.

SCOTT, J., COWELL, J., ROBERTSON, M.E. & 8 others (1985). Insulin like growth factor-II gene expression in Wilms' tumour and embryonic tissues. *Nature*, **317**, 260.

SHEA, T.C., KELLY, S.L. & HENNER, W.D. (1988). Identification of an anionic form of glutathione transferase present in many human tumors and human tumor cell lines. *Cancer Res.*, **48**, 527.

SIEGERS, C.P., BOSE-YOUNES, H., THIES, E. & YOUNES, M. (1984). Glutathione and glutathione dependent enzymes in the tumourous and nontumorous mucosa of the human colon and rectum. *J. Cancer Res. Clin. Oncol.*, **107**, 238.

SINGH, S.V., LEAL, T., ANSARI, G.A.S. & AWASTHI, Y.G. (1987). Purification and characterisation of glutathione S-transferases of human kidney. *Biochem. J.*, **246**, 179.

TEW, K.D., CLAPPER, M.L., GREENBERG, R.E., WEESE, J.L., HOFFMAN, S.J. & SMITH, T.M. (1987). Glutathione S-transferases in human prostate. *Biochim. Biophys. Acta*, **926**, 8.

WOLF, C.R., LEWIS, A.D., CARMICHAEL, J. & 7 others (1987). Glutathione S-transferase expression in normal and tumour cells resistant to cytotoxic drugs. In *Glutathione S-transferases and Carcinogenesis*. Mantle, T.J., Pickett, C.B. & Hayes, J.D. (eds). Taylor and Francis: London.